# Editorial: Designing Self-Organization in the Physical Realm

**DOI:** 10.3389/frobt.2020.597859

**Published:** 2020-11-02

**Authors:** Heiko Hamann, Melanie Schranz, Wilfried Elmenreich, Vito Trianni, Carlo Pinciroli, Nicolas Bredeche, Eliseo Ferrante

**Affiliations:** ^1^Institute of Computer Engineering, University of Lübeck, Lübeck, Germany; ^2^Lakeside Labs GmbH, Klagenfurt, Austria; ^3^Faculty of Technical Sciences, Institute of Networked and Embedded Systems, University of Klagenfurt, Klagenfurt, Austria; ^4^Institute of Cognitive Sciences and Technologies, Italian National Research Council, Rome, Italy; ^5^Robotics Engineering, Worcester Polytechnic Institute, Worcester, MA, United States; ^6^Institut des Systèmes Intelligents et de Robotique, Université Pierre et Marie Curie, Paris, France; ^7^Department of Computer Science, Vrije Universiteit Amsterdam, Amsterdam, Netherlands

**Keywords:** self-organization, cyber-physical system, swarm robotics, resilience, scalability

The design and deployment of decentralized systems can benefit from self-organization as it introduces key features, such as resilience, scalability, and adaptivity to dynamic environments. However, whenever self-organization was demonstrated on physical platforms (e.g., robot swarms), this was performed mostly within controlled laboratory conditions. The real world comes with severe requirements, calling for robust design methodologies, their standardization, and validation via benchmarking toolsets. With this Research Topic, we collect, benchmark, and survey novel approaches to push self-organization toward real-world applications, focusing on embodied artificial systems, such as multi-robot, cyber-physical, and socio-technical systems.

We start with six perspective and survey papers that give a good overview of the state of the art and challenges of real-world implementations.

Gershenson studies the complexity of cyber-physical systems. After reviewing basic concepts that are useful to design self-organizing systems, he introduces approaches to implement self-organization in cyber-physical systems. Gershenson reviews three case studies from different domains. Crowd control is related to a passive control approach using signs to mediate passenger boarding and descent in Mexico City Metro. In a traffic light case study, traffic lights and vehicles interact closely as agents, resulting in a network of streets and crossings with self-organized coordination of traffic flows. The third case study is related to public transport and addresses the equal headway instability. Trains use bio-inspired pheromone systems to keep equal distance to the vehicles in front and behind. The result is a flexible system where trains can quickly adapt and respond to service delays. Gershenson provides an outlook for cyber-physical and cyber-social systems controlled by guided self-organization.

Based on the above-mentioned benefits of self-organization the motivation is strong to apply swarm robotics in industrial applications. However, many industrial applications still rely on centralized control. In cases where a multi-robot solution is employed, the main idea of swarm robotics of distributed decision-making is often not implemented. Schranz et al. provide a collection and categorization of swarm robotic behaviors. The paper gives a comprehensive overview of research platforms and industrial projects and products, separated into terrestrial, aerial, aquatic, and outer space. The authors identify several open issues including dependability, emergent characteristics, security and safety, and communication as hindrances for the implementation of fully distributed autonomous swarm systems.

To deploy swarm robots to the physical realm, one requirement is the ability to cope with environments that lack human infrastructures. Two key mechanisms, namely cognition and sensing, have to take place “on-board” on the robot and should not be offloaded to external devices. Physical mobile robots that operate on land do have the required hardware capabilities for onboard computation and sensing, and have successfully been used to demonstrate basic collective behaviors and to a more limited extent been used in real applications. However, Coppola et al. convincingly argue that swarm robotics approaches so far cannot be applied to Micro Aerial Vehicles (MAVs). The most impressive MAVs demonstrations have been executed requiring external computation, sensing, or both. The main challenge is related to local sensing, which they divide into the following sub-challenges: MAV hardware design, ego-state estimation, intra-swarm relative sensing, and swarm behaviors. This paper presents how advanced we are in terms of autonomy of swarms of MAVs, and presents a roadmap to overcome the challenges in the near future.

One of the main challenges for the design of self-organizing systems is the gap between the rules followed by individual system components and the desired collective behavior of the system as a whole. Especially for practical application scenarios, it is difficult to conceive and optimize the system behavior by acting at the level of the individual rules. The paper by Birattari et al. champions a methodology that optimizes the system behavior offline (e.g., in simulation) and that ensures sufficient performance when deployed in the real world. The central aspect is the “class of interest” of the problems to be addressed. Every new problem instance is sampled from the same class of interest (e.g., gardening with robot swarms), and the solution is optimized to maximize performance, according to relevant metrics defined for the given class. It is within the same class of interest that the offline automatic design approach gives its best results, and the manifesto highlights the most important questions that should drive future research in this area.

The following eight papers study concepts, methods, hardware designs, and natural systems with high potential to support future real-world applications of self-organizing systems.

There have been many contributions using either simulation or relatively simple robots, often in controlled environments of limited size. Tarapore et al. question the very definition of swarm robotics by focusing on the question of how sparse is a robot swarm for a realistic task. Tarapore et al. argue that real swarm robotics applications will need to be addressed, and they introduce the idea of “sparse swarm robotics”: robots are spread over the environment such that the opportunity for communication must be explicitly addressed, as opposed to being naturally forced in smaller environment where density is high. They propose a clean and straight-forward formalization of this problem in mathematical terms. Also, they illustrate the concept of sparse swarm robotics by describing several realistic problems and their implications, including a step-by-step description of the specific issues that arise for one such problem. Considering a monitoring task for soil sampling in a forest, they discuss both low-level hardware issues and high-level communication/coordination issues.

A particular threat for real-world robot swarms is a possible attack by malicious agents that could be introduced into the swarm. The paper by Strobel et al. makes a significant contribution toward the use of swarm robotics in the real world by presenting a framework for a secure decentralized database. The presented framework uses smart contracts, a way to decentrally execute programs based on an Ethereum blockchain. Individual malicious robots aim to disrupt the collective decision-making process of a simulated swarm of e-puck robots by spreading misinformation. The robot swarm successfully disregards the wrong information. The authors indicate that blockchain networks can be used for robot swarms, and the low processing and memory capacity of swarm robots does not prohibit the use of blockchains in real-world scenarios.

When developing the swarm robot controller and hardware, it is difficult to anticipate all future situations that this robot swarm may experience. Hunt claims that nature provides an example solution that we can follow: phenotypic plasticity. The idea is to train robot swarms in (simulated) heterogeneous environments, for example, using methods of evolutionary computation. The general swarm robot design should allow for flexibility such that they can be adapted and shaped ideally in three dimensions: behavioral, physiological, and morphological plasticity. Behavioral plasticity of the swarm members introduces diversity that can be exploited, for example, to increase fault tolerance and decision accuracy. Physiological plasticity in robots could be modes of operation that have different energy consumption. Morphological plasticity could be known implementations of self-assembling swarm robots. In summary, Hunt opens a door to more flexible and dynamic ways of drafting, developing, and optimizing robot swarms for the real world mainly based on a systematic behavioral and morphological diversity.

Rausch et al. propose an empirical case study of the impact of network topology over the spread of information in a robot swarm. Specifically, they consider the possible benefits of scale-free communication topology. They experimentally show that there is actually a trade-off in using scale-free (rather than random) topology: information spreads faster, enabling quicker reactions to changes in dynamic environments, but at the cost of a decreased stability as the emergence of consensus is hindered by communication pathways of different lengths.

To ensure a smooth transition from lab to market, it is necessary to recognize user needs and to evaluate the acceptability of robot swarms. The paper by Carrilo-Zapata et al. conducts a study against three application domains wherein robot swarms are considered as game changing tools. The mutual shaping methodology proposed entails a bi-directional knowledge exchange between swarm designers and final users, raising awareness of the possibility offered by the technology but also allowing to collect important design and interaction features that can drive the deployment. Overall, the study reveals that robot swarms can play an important role within the considered application domains, above all when they work in support of human operations, rather than as entire replacements.

Another important hurdle to deploying swarms in the physical realm is robustness. Contrary to the adage “there is safety in numbers,” robustness is not an inherent benefit of robot swarms that results from redundancy. Robustness is a challenging design goal, made complex by the interplay between the benefits of redundancy and the need for scalability. Wilson et al. argue that achieving robustness through redundancy involves a careful co-design of hardware, fabrication processes, and control software. To investigate this idea, the authors present an approach to achieving robustness that involves a novel hardware-software co-design of a modular robotic platform called “DONUts” (Deformable Self-Organizing Nomadic Units). The modules are inexpensive, flexible printed circuit boards, and designed to move as a collective through magnetic interaction. Wilson et al. study several control strategies that explore the design space of inter-module connectivity to shed light on the interplay between robustness, scalability, and controllability.

Nave et al. investigate on a biological model related to social insects—the tower building behavior of red imported fire ants. Results show that individuals moving under the influence of local attraction can form large towers. The system shows a sudden density-dependent phase transition as the attraction parameter is varied. The resulting towers of simulated agents are constantly rebuilt and move over time—a feature that has to be considered for robotic applications. There is for future robotic studies, where robots build towers out of themselves in a manner similar as the fire ants. In a real-world application, a tower of robots could be useful for seeing over obstacles, providing scaffolding for climbing, or marking a location of interest. Robotic tower-builders would need capabilities for sensing neighbors, climbing onto and off one another, and supporting appropriate loads. Building such robotic tower-builders would be an interesting step for future robotics research.

While engineers take robots to the real world to automate tasks currently done by humans or impossible for humans, biologists take robots to the real world to study animal behavior. Yang et al. study a robotics-based experimental test paradigm where a robotic replica is used to influence the behavior of Zebrafish. Two setups were studied. In the individual training condition, a single fish learned to open the correct of two doors by itself. In the social training condition, a fish observes the replica approaching both doors with the correct one opening after a certain period of time. Main contributions are the technical innovation of this robot-supported experiment and the negative result indicating that there is no improvement by social learning. Yang et al. claim that their setup can generalize to other species, such as guppies and mollies but also insects, mammals, and even invertebrates. It seems promising that with ongoing technological progress we will see more of these bio-hybrid systems with robots and animals interacting closely in the real world.

In summary, all the above papers that study an engineering approach to take self-organizing robots to the field, struggle with a technological bottleneck: local sensing, coordinated actuation, and means of communication that work reliably in field environments. This is a common challenge of robotics and will require designing smart control algorithms with minimal requirements for sensing, actuation, and communication. Common to all papers in this Research Topic are deviations between model abstractions and the physical realm. We still do not know well-enough what deviations are caused by which abstraction in swarm and multi-robot models and simulations. The intrinsic stochastic nature of self-organizing systems adds to this challenge. In future work, this will require an effort toward more robust hardware, as well as verifiable swarm and robot behaviors to achieve certification. Our Research Topic covers a wide range of fields, concepts, and methods that will hopefully help to kick our robots out of the lab, pushing toward a novel “field swarm robotics,” to establish cyber-physical systems in the wild and to design distributed systems for radically novel applications using self-organization in the physical realm.

## Author Contributions

All authors listed have made a substantial, direct and intellectual contribution to the work, and approved it for publication.

## Conflict of Interest

The authors declare that the research was conducted in the absence of any commercial or financial relationships that could be construed as a potential conflict of interest.

